# The Role of Glutathione-S Transferase in Psoriasis and Associated Comorbidities and the Effect of Dimethyl Fumarate in This Pathway

**DOI:** 10.3389/fmed.2022.760852

**Published:** 2022-02-08

**Authors:** Elena Campione, Sara Mazzilli, Monia Di Prete, Annunziata Dattola, Terenzio Cosio, Daniele Lettieri Barbato, Gaetana Costanza, Caterina Lanna, Valeria Manfreda, Ruslana Gaeta Schumak, Francesca Prignano, Filadelfo Coniglione, Fabrizio Ciprani, Katia Aquilano, Luca Bianchi

**Affiliations:** ^1^Dermatology Unit, University of Rome Tor Vergata, Rome, Italy; ^2^Italy State Police Health Service Department, Ministry of Interior, Rome, Italy; ^3^Anatomic Pathology Unit, University of Rome Tor Vergata, Rome, Italy; ^4^Anatomic Pathology, Santa Maria di Ca' Foncello Hospital, Treviso, Italy; ^5^Department of Biology, University of Rome Tor Vergata, Rome, Italy; ^6^Istituto di Ricovero e Cura a Carattere Scientifico (IRCCS) Fondazione Santa Lucia, Rome, Italy; ^7^Virology Unit, University of Rome Tor Vergata, Rome, Italy; ^8^Unit of Dermatology, Department of Surgery and Translational Medicine, University of Florence, Florence, Italy; ^9^Department of Surgical Sciences, University Nostra Signora del Buon Consiglio, Tirana, Albania

**Keywords:** comorbidities, dimethyl fumarate, glutathione-S-transferase, psoriasis, pathway

## Abstract

Psoriasis vulgaris is a chronic inflammatory skin disease characterized by well-demarcated scaly plaques. Oxidative stress plays a crucial role in the psoriasis pathogenesis and is associated with the disease severity. Dimethyl fumarate modulates the activity of the pro-inflammatory transcription factors. This is responsible for the downregulation of inflammatory cytokines and an overall shift from a pro-inflammatory to an anti-inflammatory/regulatory response. Both steps are necessary for the amelioration of psoriatic inflammation, although additional mechanisms have been proposed. Several studies reported a long-term effectiveness and safety of dimethyl fumarate monotherapy in patients with moderate-to-severe psoriasis. Furthermore, psoriasis is a chronic disease often associated to metabolic comorbidities, as obesity, diabetes, and cardiovascular diseases, in which glutathione-S transferase deregulation is present. Glutathione-S transferase is involved in the antioxidant system. An increase of its activity in psoriatic epidermis in comparison with the uninvolved and normal epidermal biopsies has been reported. Dimethyl fumarate depletes glutathione-S transferase by formation of covalently linked conjugates. This review investigates the anti-inflammatory role of dimethyl fumarate in oxidative stress and its effect by reducing oxidative stress. The glutathione-S transferase regulation is helpful in treating psoriasis, with an anti-inflammatory effect on the keratinocytes hyperproliferation, and in modulation of metabolic comorbidities.

## Introduction

Psoriasis vulgaris is a chronic inflammatory skin disease characterized by well-demarcated erythema and scaly plaques. It is reported that an enhanced oxidative stress is associated with the severity of psoriasis (1). Karabowicz et al. investigated the intensity of oxidative stress and the expression and activity of the proteasomal system, as well as the autophagy, responsible for the degradation of oxidatively modified proteins in the blood cells of patients with psoriasis (2). Oxidative-antioxidant system plays a crucial role in the psoriasis pathogenesis (3). Numerous studies reveal significantly increased levels of oxidative stress markers, as malondialdehyde, nitric oxide end products, and 8-hydroxy-2′ -deoxyguanosine in the plasma of psoriatic patients. Meanwhile, a decreased total antioxidant capacity, reduced vitamin A and E levels, and a diminished activity of the main antioxidant enzymes were also detected in these patients (4). The antioxidant system involved in oxidative stress reduction is constituted by the glutathione-S transferase (GST). An increased reactive oxygen species (ROS) and insufficient antioxidant activity have been detected in psoriatic lesions (5). Pro-inflammatory cytokines are involved in redox skin balance perturbation in patients with psoriasis (3). Dimethyl fumarate (DMF) and its metabolite monomethyl fumarate (MMF) modulate some signaling proteins activity and intracellular concentration, such as the nuclear factor (erythroid-derived 2)-like 2 (Nrf2), nuclear factor-kappa B (Nf-κB), and cyclic adenosine monophosphate. Some studies showed that DMF can also affect the hypoxia-inducible factor-1 alpha. These actions seem to be responsible for i) the downregulation of inflammatory cytokines and ii) an overall shift from a pro-inflammatory (Th1/Th17) response to an anti-inflammatory/regulatory (Th2) response. Both steps are necessary for the amelioration of psoriatic inflammation, although additional mechanisms have been proposed. There is a growing body of evidence to support the notion that DMF/MMF may also exert effects on granulocytes and non-immune cell lineages, including keratinocytes and endothelial cells. A better understanding of the multiple molecular mechanisms involved in the cellular action of fumaric acid esters (FAEs) will help to adapt and to further improve the use of such small molecules for the treatment of psoriasis and other chronic inflammatory diseases (6). Superoxide dismutase (SOD) and glutathione peroxidase (GP) activity in erythrocytes are involved in the psoriasis onset (7). Imbalance in the oxidant-antioxidant system in psoriasis is involved. The DMF is considered as a prodrug, after oral administration, rapidly hydrolyzed by esterases in the small intestine and converted to MMF representing an intermediate of tricarboxylic acid cycle (TCA) (7). This molecule has been successfully used in psoriasis treatment for more than 40 years. Several clinical trials have demonstrated the FAEs efficacy in this role (6, 8). In 1994, a mixture of MMF and DMF (Fumaderm®) was approved for the oral treatment of psoriasis in Germany, Switzerland, and Austria (9). In 2019, DMF was approved for the treatment of mild-to-moderate plaque psoriasis. Several studies reported a long-term effectiveness and safety of DMF monotherapy in patients with moderate-to-severe psoriasis (9). In humans, people with polymorphisms in GST genes were described to be susceptible to various disorders, including psoriasis (10, 11), coronary artery diseases (12), chronic obstructive pulmonary diseases (13), rheumatoid arthritis (14), or neoplastic diseases, as breast, esophageal, and gastric cancers (15, 16). Furthermore, psoriasis is a chronic disease often associated with metabolic comorbidities, as obesity, diabetes, and cardiovascular diseases, wherein GST deregulation is present (17). Environmental and genetic risk factors have been implicated in obesity etiopathology (18). Also, the oxidative stress could lead to obesity, and the related comorbidities, by promoting a white adipose tissue deposition (19). Several *in vitro* studies documented that an increased oxidative stress and an ROS could augment adipocyte proliferation, differentiation, and growth (20–22), and control hunger and satiety behaviors (23). Interestingly, there is a mutual relation between oxidative stress and obesity, as abnormal fat accumulation can stimulate a pro-inflammatory and a pro-oxidant state through various biochemical and cellular mechanisms (24–26). The GST, which removes the electrophilic compounds, including the lipid peroxidation products, showed a white adipose tissue-specific downregulation (26). Additionally, the antioxidant enzyme activities of GP and superoxide dismutase were reported to be dysregulated in red blood cells and serum of obese individuals compared to controls (27, 28). Enzyme-converting glutathione is constitutionally expressed by keratinocytes (29). An increase of GST activity in psoriatic epidermis in comparison with uninvolved and normal epidermal biopsies has been reported. The DMF depletes glutathione by formation of covalently linked conjugates. Consequently, oxidized glutathione is converted to a reduced glutathione and is also depleted by DMF (30). The GST includes glutathione enzyme catalyzing conjugation with various hydrophobic compounds (29). Many data evaluated the role of conjugating activity of hydrophobic molecules, such as bilirubin and hematin linkage and selenium-independent GP activity, toward organic hydroperoxides in the oxidative stress cycle (31).

This review investigates the anti-inflammatory role of DMF in oxidative stress and its effect by reducing ROS through glutathione modulation. The GST regulation is helpful in treating psoriasis, with anti-inflammatory effect on the keratinocytes hyperproliferation and in modulation of metabolic comorbidity.

## DMF Antioxidant Activity

**Figure 1:**
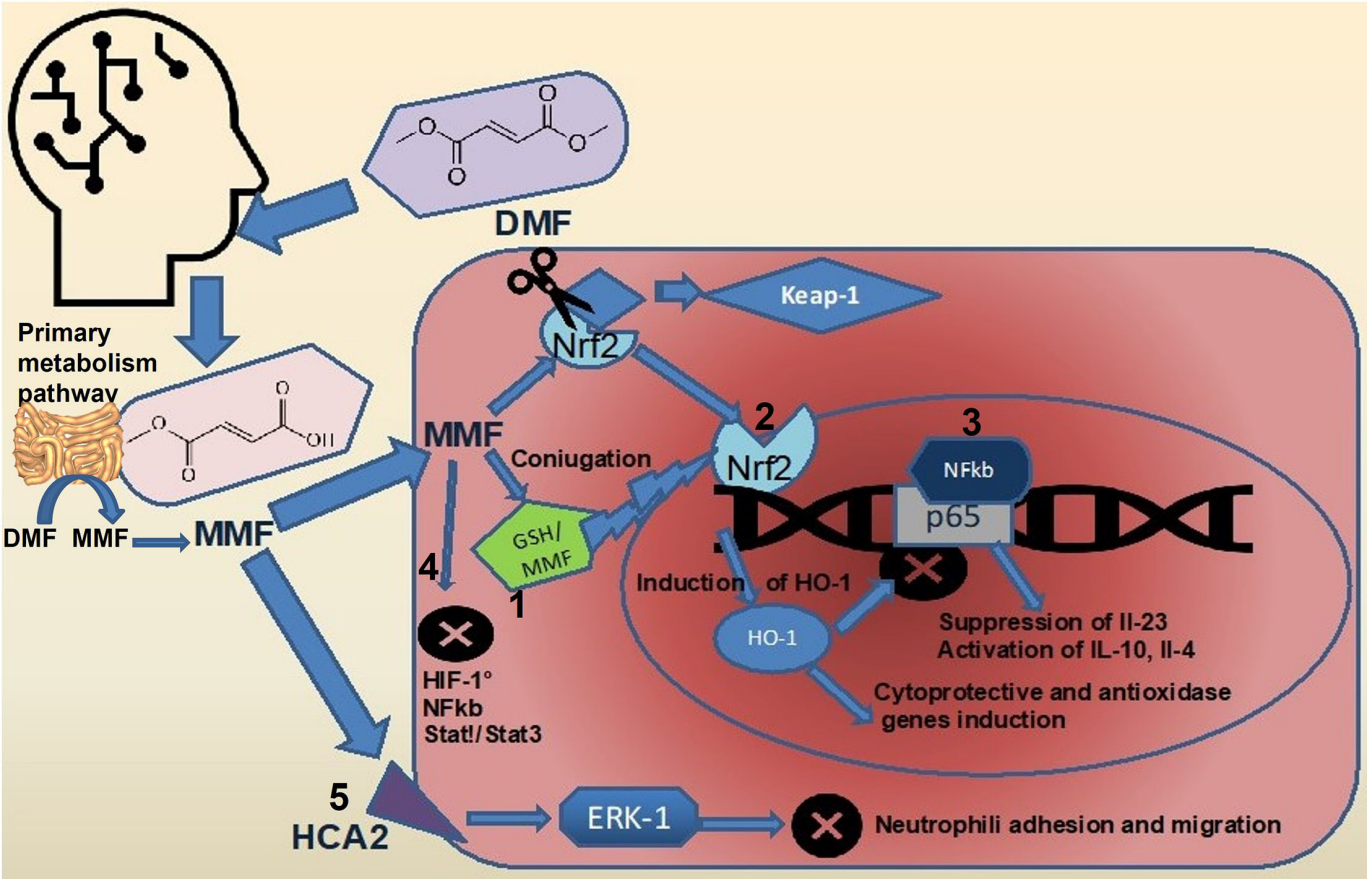
Effects of DMF on intracellular signaling pathways. Upon ingestion, most of the DMF (about 90%) is rapidly converted to MMF by hydrolization in the small intestine (5). The full pharmacokinetic profile of DMF and MMF remains to be elucidated. DMF, dimethyl fumarate; MMF, monomethil fumarate; FAEs, fumaric acid esters; GP, glutathione peroxidase; GST, glutathione-S transferase; NF-kappaB, nuclear factor-kappa B; Nrf2, nuclear factor (erythroid-derived 2)-like 2; ROS, reactive oxygen species; and TCA, tricarboxylic acid cycle.

Dimethyl fumarate (DMF) is considered a prodrug as, after oral administration, it is rapidly hydrolyzed by esterases in the small intestine and converted to MMF (32). The MMF is highly bioavailable and is rapidly hydrolyzed inside cells to fumaric acid, which in mitochondria, represent an intermediate of TCA (33, 34). It is mostly believed that DMF exerts its therapeutic effects through antioxidant and anti-inflammatory pathways (Figure 1). Both MMF and fumarate are believed to be responsible for the primary therapeutic effects of DMF through activation and inhibition of the transcription factors, Nrf2 (35, 36) and Nf-κB (37), respectively. It has been well-described that DMF activates the Nrf2 signaling pathway through the electrophilic modification of Kelch-like ECH-associated protein 1 (35). The DMF exerts its immuno-modulatory activity also *via* the agonism of the hydroxycarboxylic acid receptor 2 (38). Such important mechanisms, nonetheless, fail to fully account for the *in vitro* and *in vivo* immunologic actions of DMF (39). Recent evidence has suggested that modulation of innate and adaptive immune processes is Nrf2 independent (40). Some of the neuro-protective effects seen with this drug are secondary to its anti-inflammatory and antioxidant actions and appear to rely on the modulation of cellular metabolism. Accordingly, a short-term DMF treatment of an oligodendrocyte cell line did not prevent a hydrogen peroxide-mediated death, and a DMF treatment in a model of toxic demyelination was not able to prevent demyelination (41). Importantly, methylated esters of TCA intermediates, such as DMF, are cell permeable and can modify the activity of this pathway by increasing the level of metabolic intermediates' proximate to fumarate. In the TCA, succinate is oxidized to fumarate and then hydrated to malate through the activity of two enzymes, succinate dehydrogenase, and fumarase. Administration of DMF *in vitro* causes a rise in the concentration of succinate (42, 43). Prolonged treatment with DMF in a human oligodendrocyte cell line elicited increases in both succinate and fumarate (44). This event is associated with augmented lipid synthesis, thus, preserving mature oligodendrocytes viability, and protecting myelin through the modulation of cellular lipid metabolism. These data were confirmed *in vivo* by using global metabolomics profiling of blood plasma of patients with relapsing-remittent multiple sclerosis treated for 6 weeks with DMF. Significant changes in TCA intermediates fumarate and succinate, and in the secondary TCA metabolites succinyl-carnitine and methyl succinyl-carnitine were observed, arguing that the potential anti-inflammatory properties of these metabolites are mediated by metabolic rewiring. Interestingly these changes were not observed in the control population (45). A metabolic switch toward aerobic glycolysis is mandatory for immune cells activation. Impinging a metabolic rewiring toward mitochondrial oxidative metabolism is considered a valid strategy to counteract the inflammatory process in immune diseases (46). The DMF was shown to covalently modify protein cysteine residues in a process termed succinylation. In activated myeloid and lymphoid cells, DMF was able to downregulate aerobic glycolysis *via* the succinylation and inactivation of the glycolytic enzyme glyceraldehyde 3-phosphate dehydrogenase, thereby inhibiting the autoimmune response (47). Immune cell activation also depends on calcium signaling. Among the proposed mechanisms for the immunoregulatory role of DMF, the rise of intracellular calcium is also included. In particular, DMF promotes an immediate extracellular calcium influx, long-term increase of cytosolic calcium, and reduced intracellular calcium storage. Upon DMF treatment, the glutathionylation of a cysteine of sarco/endoplasmic reticulum Ca^2+^-ATPase SERCA2b is critical to the modulation of intracellular calcium concentration. The SERCA2b is downregulated but more active due to glutathionylation of the redox-sensitive cysteine. A net increase of cytosolic calcium due to a diminished calcium storage is, therefore, obtained (48). Fumarate also functions as an immuno-modulator by controlling chromatin modifications. Fumarate can also rewire the epigenetic landscape of the cells through inhibiting either histone or DNA demethylases. Fumarate accumulation has been demonstrated in activated immune cells, and this event inhibits KDM5 histone demethylase activity, thus, promoting the transcription of promoters of TNF-α and IL-6 cytokines (49). Upon DMF treatment, different proteins in T cells are susceptible to covalent modifications of cysteines. Protein kinase C θ modification avoids its association with the co-stimulatory receptor CD28, preventing a T-cell activation (50). Besides such immuno-modulatory actions, DMF has an important antioxidant activity; the way by which it reduces oxidative stress is very peculiar. Actually, it scavenges the major intracellular non-enzymatic thiol antioxidant glutathione (51–54), likely, *via* the immediate formation of glutathione-DMF adducts (55), and this results in the stabilization and in the raise of Nrf2. Nrf2 then translocates into the nucleus and binds to antioxidant response elements in the promoter region of several antioxidant genes, such as heme-oxygenase-1 and NADPH-quinone-oxidoreductase-1. This, in turn, increases the intracellular concentration of glutathione (35, 56), making the cell more resistant to oxidative stress. However, DMF is able to raise glutathione levels also when the rate-limiting enzyme of glutathione synthesis, i.e., glutamate-cysteine ligase, is inhibited, thanks to the Nrf2-mediated induction of glutathione reductase that enhances the molecule recycling (57).

## The Role of Glutathione and DMF in Psoriasis

Several studies have demonstrated that glutathione binding to DNA is able to regulate Nf-κB proinflammatory activity. In particular, the Nf-κB complex and the upstream proteins, as TRAF6, are negatively regulated by glutathione (58). Genetic polymorphisms affecting GST produce a decrease in intracellular concentration of glutathione, with consequent raising of skin inflammation, as seen in atopic and allergic dermatitis, psoriasis, lichen planus, urticaria, and vitiligo (59–62).

Glutathione plasmic levels and GP activity in patients with psoriasis were significantly lower than in general population (63). Consequently, GST activity reduction leads to the accumulation of ROS in inflamed lesions, as it was reported in psoriatic plaques, where ROS levels are 3-fold higher than in non-lesioned skin (64). The DMF action in this context is not yet completely clear. It irreversibly binds the glutathione in a 1:1 ratio, decreasing its production and favoring its excretion through urine as glutathione-DMF adducts (65). In this way, fumarate compounds influence cellular redox state, affecting intracellular signaling pathways (66).

Glutathione intracellular depletion in human antigen-presenting cells causes IL-10 production, with immuno-modulatory action, instead of the pro-inflammatory cytokines IL-12 and IL-23, responsible for Th1/Th17 immune system response switch in psoriasis. In this context, DMF promotes Th2 cell differentiation, with immunoregulatory functions (67).

In summary, the rationale of employing DMF in psoriasis consists in reducing cellular inflammation both by decreasing glutathione intracellular levels and by inducing a switch in immune response toward an anti-inflammatory/immunoregulatory setting (68, 69). European guidelines recommend FAEs in induction and long-term therapy of moderate-to-severe plaque psoriasis (70). With more than 220,000 patients per year treated with FAEs, Germany has been one of the first nations in Europe to adopt this systemic therapy for psoriasis (71), but also other countries, like Italy, are aligned with European guidelines (7). The recommendation in the treatment with DMF is to begin with a low dose followed by gradual increases. This flexible approach is tailored on the need of each patient, and the most used regimen is between 240 mg and 480 mg of DMF per day. Several randomized clinical trials have demonstrated efficacy and safety of FAEs in psoriasis. At week 16 of the phase III, randomized, BRIDGE study, PASI 75 was reached by more than one third of patients enrolled (8), while in the large retrospective FUTURE study it was demonstrated a mean reduction of 79% in PASI from baseline (72). Combination of topical treatments, biological agents, or phototherapy to FAEs in the induction phase showed to reach a faster response (73–75). The FAEs are also characterized by a mild spectrum of side effects, including gastrointestinal disorders and flushing during the treatment, which are not responsible for therapy discontinuation. Among the others, the most important is lymphopenia, which is, generally, of a mild entity and experienced during induction or when it is necessary to increase the dose regimen. It is necessary in such cases to adjust the dosage at the higher tolerance. Treatment discontinuation is required only in rare cases to minimize opportunistic infections' risk (76).

## The Role of Small Molecules in the Metabolic Syndrome

Patients with psoriasis are characterized by a higher prevalence of cardiovascular disease and metabolic syndrome (77). In particular, visceral fat has a critical role in the development of cardiovascular disease in patients with psoriasis, including coronary arteries disease, heart infarction, stroke, and related mortality. Moreover, the inflammatory background of the patients with psoriasis both increases and accelerates the atherosclerosis (77). Small molecules, as the phosphodiesterase-4 inhibitor apremilast, approved for the treatment of adults with moderate-to-severe plaque psoriasis and/or psoriatic arthritis, have demonstrated a broad anti-inflammatory activity, which may influence metabolism (17, 78). It has been demonstrated that liver steatosis is reduced by limiting the fat deposition and increasing lipolysis (17). The patients with diabetes reached better results in terms of psoriasis response when treated with apremilast. Moreover, it was observed as a better control of serum glucose levels, a significant reduction of insulin resistance and cholesterol levels, and the restoration of endothelial function, which are all factors strongly associated with propensity to cardiovascular diseases. Finally, apremilast also decreases the systemic inflammatory status of patients with psoriasis, decreasing TNF-α, IFN-γ, IL-12, and IL-23 production (17). As an apremilast, DMF also exhibits strong anti-inflammatory and immunomodulatory effects and was tested in a laboratory to evaluate its role in ameliorating basal inflammation and metabolic disturbances (79). Compared to control rats, those treated with FAEs showed lower levels of C-reactive protein, IL-6, and TNF-α. Moreover, it was demonstrating less fat accumulation, with lower visceral fat weight in liver and muscles. These results suggest the potential crucial role of DMF, as an apremilast, in the treatment of patients with psoriasis with concurrent metabolic comorbidities, which are probably the largest part.

## Author Contributions

Conceptualization was contributed by EC, SM, MD, AD, TC, DL, GC, CL, VM, RG, FP, FCo, KA, and LB. Methodology was contributed by EC, MD, AD, TC, DL, GC, CL, VM, RG, FP, FCo, FCi, KA, and LB. Validation was contributed by EC, SM, MD, TC, DL, GC, CL, VM, RG, FP, FCo, FCi, KA, and LB. Formal analysis was contributed by EC, SM, MD, AD, DL, GC, CL, VM, RG, FP, FCo, KA, and LB. Investigation was contributed by EC, SM, MD, AD, TC, GC, CL, VM, RG, FP, FCo, KA, and LB. Resources was contributed by EC, SM, MD, AD, TC, DL, CL, VM, RG, FP, FCo, KA, and LB. Data curation was contributed by EC, SM, MD, AD, TC, DL, GC, VM, RG, FP, FCo, KA, and LB. Writing—original draft preparation was contributed by EC, SM, MD, AD, TC, DL, GC, CL, RG, FP, FCo, KA, and LB. Writing—review and editing were contributed by EC, SM, MD, AD, TC, DL, GC, CL, VM, FP, FCo, FCi, KA, and LB. Visualization was contributed by EC, SM, MD, AD, TC, DL, GC, CL, VM, RG, FCo, KA, and LB. Supervision was contributed by EC, SM, MD, AD, TC, DL, GC, CL, VM, RG, FP, FCi, KA, and LB. Project administration was contributed by EC, SM, MD, AD, TC, DL, GC, CL, VM, RG, FP, FCo, and LB. All authors approved the submitted version and agreed to be personally accountable for the author's own contributions and for ensuring that questions related to the accuracy or integrity of any part of the work, even ones in which the author was not personally involved, are appropriately investigated, resolved, and documented in the literature.

## Funding

This study received funding from Almirall S.p.A. The funder was not involved in the study design, collection, analysis, interpretation of data, the writing of this article or the decision to submit it for publication.

## Conflict of Interest

The authors declare that the research was conducted in the absence of any commercial or financial relationships that could be construed as a potential conflict of interest.

## Publisher's Note

All claims expressed in this article are solely those of the authors and do not necessarily represent those of their affiliated organizations, or those of the publisher, the editors and the reviewers. Any product that may be evaluated in this article, or claim that may be made by its manufacturer, is not guaranteed or endorsed by the publisher.
